# The SGK3-triggered ubiquitin–proteasome degradation of podocalyxin (PC) and ezrin in podocytes was associated with the stability of the PC/ezrin complex

**DOI:** 10.1038/s41419-018-1161-1

**Published:** 2018-11-01

**Authors:** Ya-Pei Yuan, Hong Zhao, Li-Qin Peng, Zi-Fang Li, Song Liu, Cheng-Yan Yuan, Mercy-Julian Mwamunyi, David Pearce, Li-Jun Yao

**Affiliations:** 1 Department of Nephrology, Union Hospital, Tongji Medical College, Huazhong University of Science and Technology, 430022 Wuhan, China; 2 Department of Trauma Surgery, Tongji Hospital, Tongji Medical College, Huazhong University of Science and Technology, 430030 Wuhan, China; 3 Department of Rheumatology, Sun Yat-sen Memorial Hospital, Sun Yat-sen University, 510120 Guangzhou, China; 4 Department of Medicine, University of California, San Francisco, CA 94107-2140 USA; 5 Department of Molecular and Cellular Pharmacology, University of California, San Francisco, CA 94107-2140 USA

## Abstract

Podocyte damage is commonly accompanied by destabilization of the podocalyxin (PC)/ezrin complex. Serum- and glucocorticoid-inducible kinase 3 (SGK3) plays a role in the maintenance of podocyte function, but the details of this role are poorly understood. Herein we demonstrated that SGK3 and its downstream target protein neural precursor cell expressed developmentally downregulated protein 4 subtype 2 (Nedd4-2) triggered PC and ezrin interaction. In adriamycin (ADR)-induced nephritic mice, and after puromycin aminonucleoside (PAN)-induced podocyte damage in vitro, PC and ezrin protein expression levels decreased significantly, while Nedd4-2 activity increased. Moreover, PAN treatment increased PC and ezrin ubiquitination and decreased PC/ezrin interaction in cultured mouse podocytes. The downregulation of SGK3 activity in mouse podocytes resulted in decreased PC and ezrin protein expression and increased the ubiquitin–proteasome degradation of PC and ezrin. Furthermore, upregulation of SGK3 activity mostly reversed the PAN-induced decrease in PC and ezrin protein expression. Overexpression of Nedd4-2 led to decreased ezrin protein expression via the upregulation of ezrin ubiquitination. In contrast, Nedd4-2 knockdown resulted in increased ezrin protein expression but decreased ezrin ubiquitination. In PC-transfected human embryonic kidney (HEK293T) cells, SGK3 activity downregulation and Nedd4-2 overexpression resulted in decreased PC/ezrin interaction. These results suggested that SGK3 triggers the ubiquitin–proteasome degradation of PC and ezrin, while the SGK3/Nedd4-2 signaling pathway regulates ezrin, but not PC, ubiquitination. Thus SGK3 helps to regulate podocyte function by maintaining the stability of the PC/ezrin complex.

## Introduction

Proteinuria, an important clinical manifestation of chronic kidney disease (CKD), may be caused by the loss of podocyte structural protein function^1–3^, leading to glomerular sclerosis and end-stage renal failure. In order to understand pathogenesis of proteinuria and to design therapeutic strategies to prevent the development of proteinuric CKD, investigations of the signaling pathways underlying podocyte damage are necessary.

We previously reported that a lack of serum- and glucocorticoid-inducible kinase 3 (SGK3) resulted in proteinuria in mice and that SGK3 activity and expression were inhibited after podocyte damage, both in vivo and in vitro^4^. Other studies have shown that the downstream target protein of SGK3, glycogen synthase kinase-3 (GSK3), is involved in the regulation of podocytosis, proteinuria, and podocyte autonomic injury^5,6^. Like AKT (also known as protein kinase B), SGK3 is a member of the serine–threonine protein kinase family and is highly expressed in mammalian kidney tissue^7^. Extensive studies have shown that AKT2 regulates the survival and function of podocytes during CKD^8–10^. AKT2 and SGK3 are fairly homologous in structure and function^11^. Thus it is probable that SGK3 is involved in the pathogenesis of proteinuria after podocyte damage^4^. However, the mechanism by which SGK3 induces podocyte dysfunction remains poorly understood.

Podocalyxin (PC) is a heavily sialylated and sulfated membrane protein expressed on the apical surface of glomerular epithelial cells (podocytes)^12,13^. PC is composed of a mucin domain, a disulfide-bonded globular domain, a transmembrane region, and a highly charged cytoplasmic tail that has potential phosphorylation sites for protein kinase C and casein kinase II^14^. This highly conserved cytoplasmic PC tail interacts with cytoskeletal actin through the Na^+^/H^+^ exchange Factor 2 (NHERF2)/ezrin complex to maintain podocyte morphology, motility, and anti-adhesion^15–18^. A number of studies have shown that urinary PC can be used as a marker for podocyte damage^19,20^. Ezrin, a member of the ezrin–radixin–moesin (ERM) protein family, maintains the stability of PC on the podocyte membrane^21,22^. The deletion of the PC/ezrin complex results in the shedding of PC from the podocyte membrane, the disappearance of the charged filtration barrier from the membrane, the fusing of the podocyte foot processes, and proteinuria^17^. Although it has been reported that the dissociation of the PC/ezrin complex leads to the disruption of foot process architecture^23,24^, the details of the molecular mechanisms connecting PC/ezrin complex disruption to podocyte damage remain unclear.

The neural precursor cell expressed developmentally downregulated protein 4 subtype 2 (Nedd4-2) protein is a downstream target of SGK3^7,25^. Nedd4-2 is a member of the homologous to E6-AP C-terminus (HECT) ubiquitin (Ub) ligase E3 family and is mainly distributed in the mammalian livers, kidneys, lungs, hearts, and brains. Nedd4-2 binds to the PPxY (PY) motif of its target proteins, recognizing and labeling target proteins with Ub to initiate ubiquitin-mediated protein degradation^26,27^. Unlike PC, ezrin has a PY motif that may bind the WW domain of Nedd4-2. However, it is unknown whether Nedd4-2 regulates the expression and function of ezrin in podocytes by mediating ezrin ubiquitination. Thus we here investigated the effects of SGK3 and the SGK3/Nedd4-2 signaling pathway on PC and ezrin ubiquitination, as well as on PC/ezrin interaction.

## Results

### PC and ezrin were downregulated in the adriamycin (ADR) mouse model

**Fig. 1:**
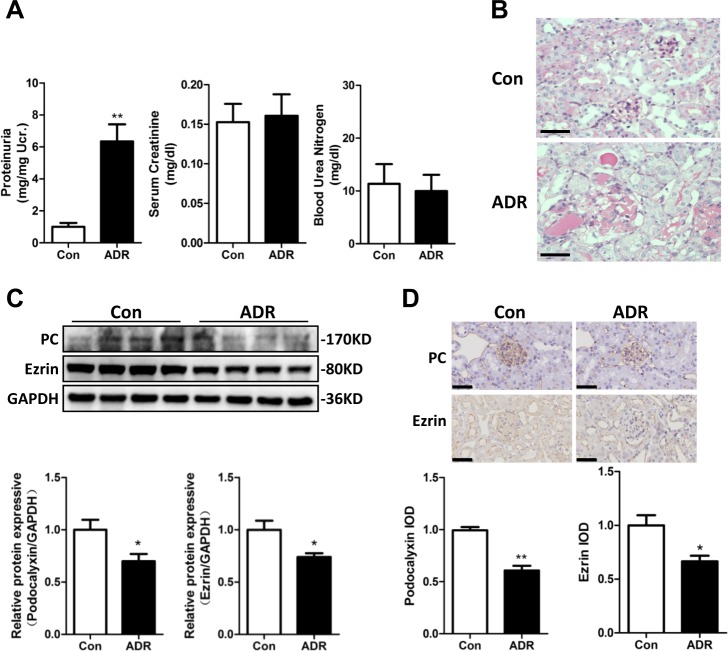
The expression levels of PC and ezrin in an ADR mouse model. An intravenous injection of ADR (10.5 mg/kg) was used to induce nephritis in the mouse model. **a** Urinary albumin levels, blood urea nitrogen and serum creatinine in mice at 14 days after ADR injection. Urinary albumin excretion were presented as mg/mg of urinary creatinine. **b** Representative images of PAS staining micrographs show kidney injury at 2 weeks after ADR injection in different groups of mice. Scale bar = 50 μm. **c** Top panel: immunoblot analysis of PC and ezrin expression in control and ADR-injected mice; GAPDH was used for normalization. Bottom panel: densitometric quantification of PC and ezrin. **d** Top panel: immunohistochemical staining shows PC and ezrin expression in the glomerulus after ADR injury. Mice were sacrificed 14 days after ADR injection, and paraffin-embedded kidney sections were stained with PC and ezrin antibodies. Bottom panel: computerized morphometric quantification of PC and ezrin staining in the glomeruli of control and ADR mice. Scale bar = 50 μm; *n* = 6/group; *P < 0.05 vs. control mice; **P < 0.01 vs. control mice

As previous studies have shown that ezrin downregulation or the inhibition of PC/ezrin complex activity can lead to podocyte dysfunction^28–30^, we first investigated whether the expression levels of PC and ezrin were altered after podocyte damage in an ADR-induced nephritic mouse model, which was established as previously described^4^. By 14 days after ADR injection, ADR-treated mice had developed significant proteinuria in comparison with the control group (Fig. 1a). Morphological kidney injuries were clearly shown by periodic acid-Schiff (PAS) staining 2 weeks after ADR injection. These injuries were characterized by glomerular sclerotic lesions and by a tubular dilation with an expanded lumen loaded with proteins (Fig. 1b). Both PC and ezrin were significantly downregulated in the kidneys of ADR mice, as compared to the control mice (Fig. 1c). PC was highly expressed in the glomerulus and peritubular capillaries of the mice, while ezrin was expressed in the glomerulus and the renal tubules. Ezrin was also known as a marker of activated or damaged podocytes in experimental models^28^. PC and ezrin expression levels, as shown by immunohistochemical staining, were significantly lower in the glomeruli of ADR mice than in the glomeruli of control mice (Fig. 1d). Thus, consistent with previous studies^30,31^, PC and ezrin were downregulated after podocyte damage in vivo.

### Puromycin aminonucleoside (PAN) treatment disrupted the PC/ezrin interaction in cultured mouse podocytes by inhibiting PC and ezrin protein expression

**Fig. 2:**
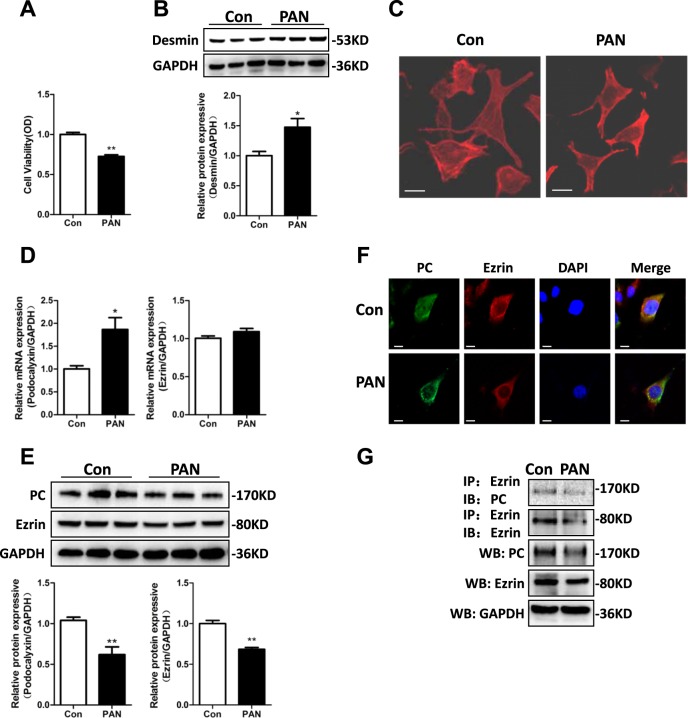
PAN inhibited PC and ezrin protein expression and downregulated PC/ezrin interaction in cultured mouse podocytes. Immortalized mouse podocytes were exposed to 50 µg/ml PAN for 24 h. **a** MTT assay was used to measure cell viability after PAN treatment for 24 h. **b** Top panel: Immunoblot analysis for desmin expression; GAPDH was used for normalization. Bottom panel: Quantification of desmin by densitometry. **c** Immunofluorescence staining showed F-actin rearrangement in PAN-treated podocytes. Scale bar = 25 μm. **d** After PAN treatment, PC and ezrin mRNA expression levels were detected with qRT-PCR. **e** Upper panel: immunoblot analysis of PC and ezrin protein expression. Bottom panel: densitometric quantification of PC and ezrin. Data are means ± SEs of three independent experiments. *P < 0.05 vs. the control group; **P < 0.01 vs. the control group. **f** Confocal immunofluorescence images show PC and ezrin staining in cultured podocytes after treatment with PAN. Scale bar = 25 μm. **g** After PAN treatment, whole-cell lysates (WCLs) were precipitated with an anti-ezrin antibody. The immunoprecipitates were analyzed with a western blot using anti-ezrin and anti-PC antibodies. PC and ezrin protein expression in WCLs was analyzed with an immunoblot

To explore PC and ezrin expression after podocyte damage in vitro, mouse podocytes were cultured and treated with 50 µg/ml PAN for 24 h. As demonstrated by 3-(4, 5-dimethyl-2-thiazolyl)−2,5-diphenyl-2-H-tetrazolium bromide (MTT) assays and western blots, PAN treatment reduced podocyte viability and increased the expression of the podocyte damage maker desmin in comparison with control cells (Fig. 2a, b). This indicated that an in vitro PAN-induced podocyte damage model had been successfully generated. In addition, F-actin was distributed as parallel bundles of stress fibers in the control podocytes; PAN treatment destroyed these stress fibers (Fig. 2c). Reverse transcriptase (RT)-PCR analysis showed that PC mRNA expression in mouse podocytes increased significantly after PAN treatment, as compared to untreated mouse podocytes, while ezrin mRNA expression was unchanged (Fig. 2d). However, PC and ezrin protein expression levels were dramatically lower in the mouse podocytes treated with PAN than in the control mouse podocytes (Fig. 2e).

Because PC and ezrin are co-expressed in podocytes, we next used confocal microscopy and co-immunoprecipitation (Co-IP) assays to test whether PAN regulated the PC/ezrin interaction. Confocal immunostaining revealed that PAN treatment decreased interaction between PC and ezrin in cultured mouse podocytes (Fig. 2f). Co-IP assays indicated that the PC/ezrin interaction in mouse podocytes treated with PAN was reduced as compared to control cells, indicating that PAN was directly involved in the co-precipitation of PC and ezrin (Fig. 2g). Our results therefore suggested that PAN treatment disrupted the stability of the PC/ezrin complex, implying that the PAN-induced decrease in PC and ezrin protein expression in mouse podocytes might not be due to the effect of PAN on PC and ezrin mRNA transcription.

### PAN increased the ubiquitin-mediated protein degradation of PC and ezrin in cultured mouse podocytes

**Fig. 3:**
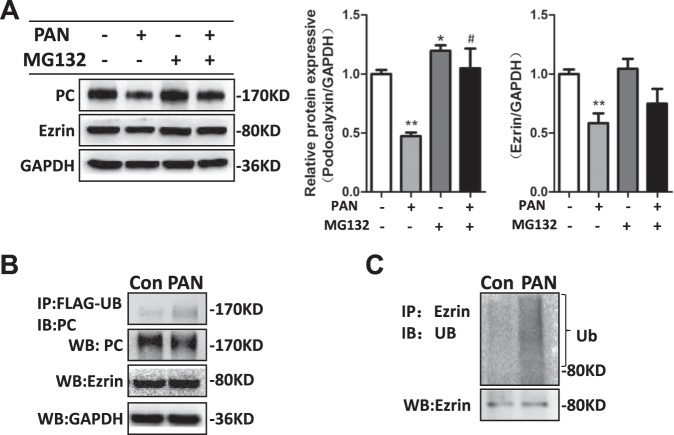
PAN enhanced the ubiquitin-mediated degradation of PC and ezrin in cultured mouse podocytes. **a** After treatment with PAN, mouse podocytes were either untreated or treated with 10 µM MG132 for the last 8 h of transfection. Left panel: immunoblot analysis showing PC and ezrin expression. GAPDH was used for normalization. Right panel: densitometric quantification of PC and ezrin. Data are means ± SEs of three independent experiments. *P < 0.05 vs. the PAN^−^/MG132^−^ cells, **P < 0.01 vs. the PAN^−^/MG132^−^ cells. ^#^*P* < 0.05 vs. the PAN^+^/MG132^−^ cells. **b** Mouse podocytes were transfected with a Ub-FLAG plasmid for 48 h and treated with PAN for the last 24 h of transfection. The proteasome inhibitor MG132 was added to the mouse podocytes for the last 8 h of transfection to prevent protein degradation. WCLs were precipitated with anti-FLAG antibodies, and the immunoprecipitates were immunoblotted with anti-PC antibodies. PC and ezrin expression was measured in WCLs after PAN treatment using immunoblotting. **c** Mouse podocytes were treated with PAN for 24 h and with MG132 for the last 8 h of PAN treatment. WCLs were precipitated with anti-ezrin antibodies, and the immunoprecipitates were immunoblotted with anti-ezrin and anti-ubiquitin antibodies

The decreased protein expression of PC and ezrin induced by PAN might be the result of increased PC and ezrin protein degradation. To test this hypothesis, we administered the proteasome inhibitor MG132 after PAN treatment to reduce ubiquitin-mediated protein degradation. When MG132 was added after PAN treatment, the protein expression levels of PC and ezrin no longer decreased (Fig. 3a). We next determined the degree of PC and ezrin ubiquitination after PAN treatment. As no commercial PC antibodies for Co-IP are yet available, we transfected mouse podocytes with a Ub-FLAG plasmid for 48 h and treated mouse podocytes with PAN for the last 24 h of transfection. The ubiquitination levels of PC (Fig. 3b) and ezrin (Fig. 3c) increased remarkably in the PAN-treated mouse podocytes as compared to the control mouse podocytes, suggesting that ubiquitin-mediated degradation of the PC and ezrin proteins is involved in PAN-mediated podocyte damage.

### SGK3-mediated PC and ezrin protein expression was involved in PAN-mediated podocyte damage

**Fig. 4:**
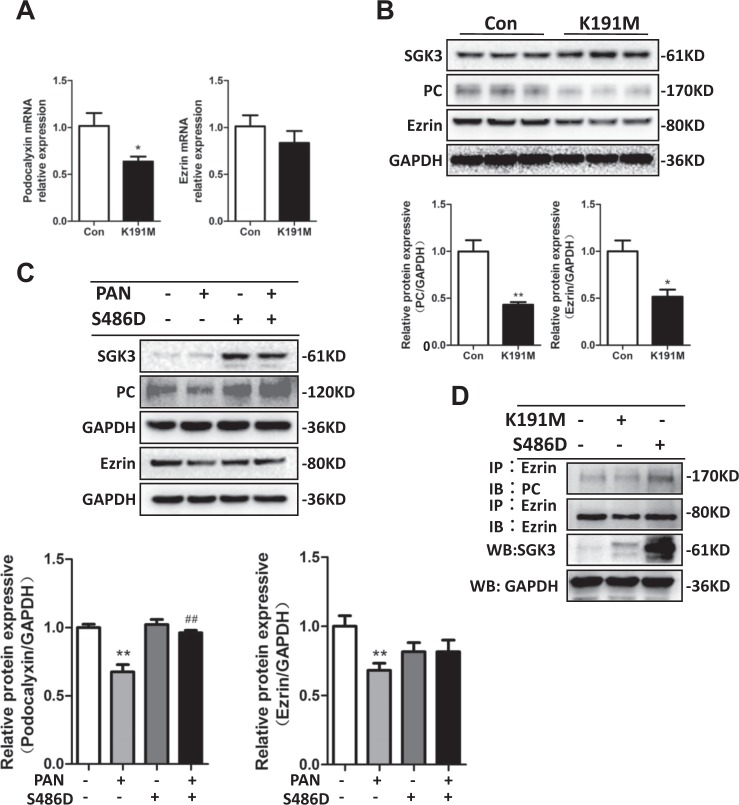
SGK3-triggered PC and ezrin protein expression was involved in PAN-induced podocyte damage. **a** The scramble (control) or SGK3-K191M plasmid were transiently transfected into cultured mouse podocytes for 48 h. qRT-PCR was used to detect the mRNA expression levels of PC and ezrin. **b** Upper panel: immunoblot of PC and ezrin expression; GAPDH was used for normalization. Bottom panel: densitometric quantification of PC and ezrin. Data are means ± SEs of three independent experiments. *P < 0.05 vs. the control group, **P < 0.01 vs. the control group. **c** The scramble (control) or SGK3-S486D plasmid were transiently transfected into cultured mouse podocytes for 48 h. The podocytes were exposed to 50 µg/ml PAN for the last 24 h of transfection. Top panel: immunoblot showing expression of SGK3, PC, and ezrin; GAPDH was used for normalization. Bottom panel: densitometric quantification of SGK3, PC, and ezrin. Data are means ± SEs of three independent experiments. **P < 0.01 vs. the PAN^−^/S486D^−^ group; ^##^*P* < 0.01 vs. the PAN^+^/S486D^−^ group. **d** Mouse podocytes were transfected with the scramble (control) plasmid, SGK3-K191M, or SGK3-S486D plasmid in vitro. WCLs were precipitated with anti-ezrin antibody, and the immunoprecipitates were immunoblotted with anti-ezrin and anti-PC antibodies. SGK3 expression was measured in WCL immunoblots after SGK3-K191M or SGK3-S486D transfection

We recently reported that SGK3 was involved in the ADR-induced nephritic mouse model and in the PAN-induced podocyte damage cell model^4^. However, it remains unclear whether SGK3 participates in the regulation of PC and ezrin protein expression. In cultured mouse podocytes transfected with an eukaryotic expression vector encoding the constitutively inactivated K191M mutant of SGK3 (SGK3-K191M), PC mRNA expression was significantly reduced as compared to control cells, but ezrin mRNA expression was unaffected (Fig. 4a). The protein expression levels of PC and ezrin in the SGK3-K191M-transfected mouse podocytes were significantly lower than the PC and ezrin expression levels in the scramble plasmid-transfected mouse podocytes (Fig. 4b). This suggested that SGK3 might be involved in the regulation of PC and ezrin expression.

To further investigate the involvement of SGK3 in the downregulation of PC and ezrin after PAN-mediated podocyte injury, cultured mouse podocytes were transfected with the constitutively activated S486D mutant of SGK3 (SGK3-S486D) or scramble plasmid for 48 h and treated with PAN for the last 24 h of transfection. We found that PC and ezrin expression levels were significantly lower in the PAN^+^/S486D^−^ transfected mouse podocytes, as compared to the PAN^−^/S486D^−^-transfected mouse podocytes. Notably, the expression levels of PC and ezrin in the PAN^+^/S486D^+^-transfected mouse podocytes were almost equivalent to those in the PAN^−^/S486D^−^-transfected podocytes, indicating that the increased activation of SGK3 mostly reversed the PAN-induced downregulation of PC and ezrin. In addition, PC expression did not differ significantly between the PAN^+^/S486D^+^- and the PAN^−^/S486D^+^-transfected mouse podocytes, suggesting that PAN treatment did not further downregulate PC protein expression in mouse podocytes transfected with constitutively activated SGK3 (Fig. 4c).

To study the role of SGK3 in the PC/ezrin complex, mouse podocytes were transfected with either the SGK3-K191M or the SGK3-S486D plasmid in vitro. PC and ezrin binding was then measured with Co-IP assays. We found that, compared with untransfected mouse podocytes, the interaction between PC and ezrin decreased in mouse podocytes transfected with SGK3-K191M but increased significantly in mouse podocytes transfected with SGK3-S486D (Fig. 4d). Therefore, ourresults indicated that SGK3 not only alleviated the PAN-induced downregulation of PC and ezrin but also maintained PC/ezrin complex stability.

### SGK3 triggered ubiquitin-mediated degradation of PC and ezrin in cultured mouse podocytes

**Fig. 5:**
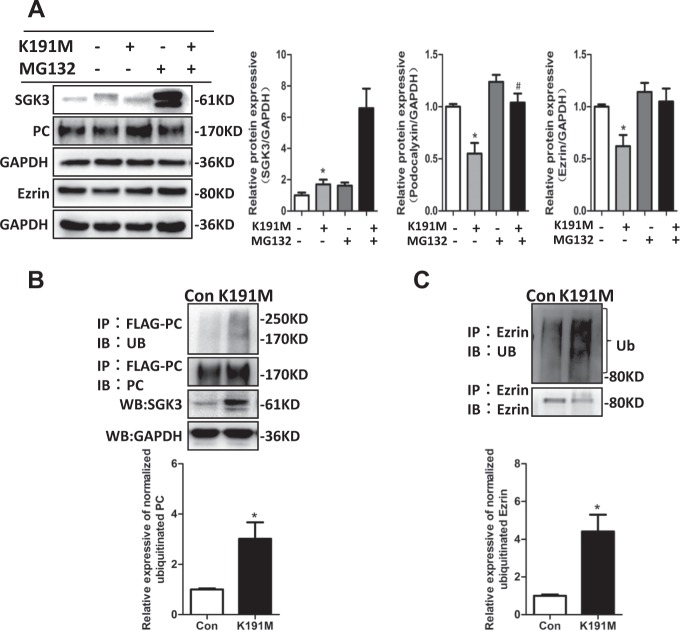
SGK3 inactivation promotes the ubiquitin-mediated degradation of PC and ezrin in cultured mouse podocytes. Mouse podocytes were transfected with the scramble (control) or SGK3-K191M plasmid. After 40 h of transfection, cells were either untreated or treated with MG132 for additional 8 h. **a** Left panel: immunoblot showing SGK3, PC, and ezrin expression; GAPDH was used for normalization. Right panel: densitometric quantification of SGK3, PC, and ezrin. Data are means ± SEs of three independent experiments. *P < 0.05 vs. the K191M^−^/MG132^−^-transfected group. ^#^*P* < 0.05 vs. the K191M^+^/MG132^−^-transfected group. **b** PC-FLAG-transfected mouse podocytes were either mock co-transfected or co-transfected with the SGK3-K191M plasmid and then treated with MG132 for the last 8 h of transfection. Upper panel: WCLs were precipitated with the anti-FLAG antibody, and the immunoprecipitates were blotted with anti-PC and anti-Ub antibodies. WCL immunoblots were used to measure SGK3 expression after SGK3-K191M transfection. Bottom panel: The level of ubiquitinated ezrin was normalized to the immunoprecipitated PC. **c** Mouse podocytes were transfected with scramble (control) or SGK3-K191M plasmid for 48 h and then treated with MG132 for the last 8 h of transfection. Upper panel: WCLs were precipitated with anti-ezrin antibody, and western blots with anti-ezrin and anti-Ub antibodies were performed after the immunoprecipitates. Bottom panel: The level of ubiquitinated ezrin was normalized to the immunoprecipitated ezrin. Data are means ± SEs of three independent experiments. *P < 0.05 vs. control group

To explore how SGK3 affected the protein expression of PC and ezrin, the SGK3-K191M plasmid was transfected into mouse podocytes to decrease SGK3 activity. The proteasome inhibitor MG132 was added for the last 8 h of transfection to inhibit protein degradation. The downregulation of PC and ezrin in SGK3-K191M-transfected mouse podocytes was mostly reversed by MG132 (Fig. 5a), indicating that SGK3 may regulate PC and ezrin expression through the proteasome pathway. Consistent with this, PC and ezrin ubiquitination levels were significantly higher in the SGK3-K191M-transfected mouse podocytes as compared to control mouse podocytes (Fig. 5b, c). These results suggested that, in the absence of SGK3 activity, PC and ezrin degradation is stimulated through the ubiquitin–proteasome degradation pathway.

### The SGK3/Nedd4-2 signaling pathway played a role in podocyte injury in vivo and in vitro

**Fig. 6:**
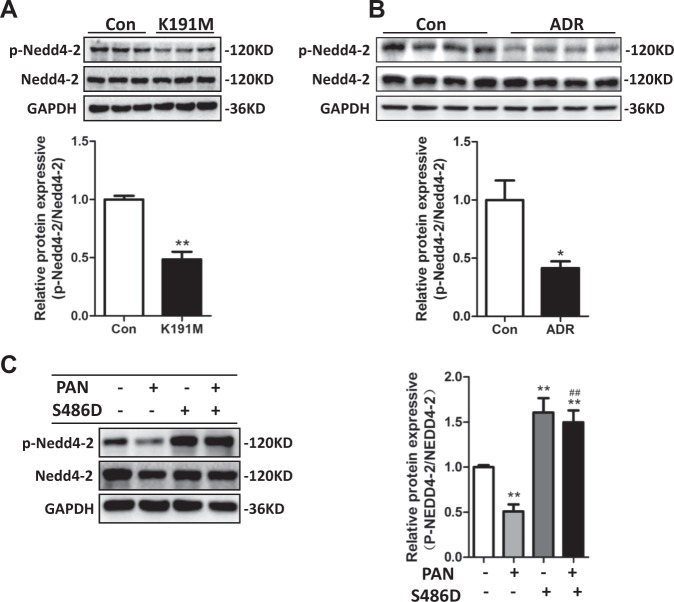
The SGK3/Nedd4-2 signaling pathway was involved in podocyte damage both in vivo and in vitro. **a** Mouse podocytes were transfected with either the scramble (control) or the SGK3-K191M plasmid for 48 h. Upper panel: immunoblot showing p-Nedd4-2 and Nedd4-2 expression; GAPDH was used for normalization. Bottom panel: densitometric quantification of the p-Nedd4-2/Nedd4-2 ratio. Data are means ± SEs of three independent experiments. **P < 0.01 vs. the control group. **b** Upper panel: immunoblot showing p-Nedd4-2 and Nedd4-2 expression in control and ADR-injected mice; GAPDH was used for normalization. Bottom panel: Densitometric quantification of the p-Nedd4-2/Nedd4-2 ratio (6 samples/group). **P < 0.01 vs. the control mice. **c** The scramble or SGK3-S486D plasmid were transiently transfected into cultured mouse podocytes. Podocytes were treated with PAN for the last 24 h of transfection. Left panel: immunoblot showing p-Nedd4-2 and Nedd4-2 expression; GAPDH was used for normalization. Right panel: densitometric quantification of the p-Nedd4-2/Nedd4-2 ratio. Data are means ± SEs of three independent experiments. **P < 0.01 vs. PAN^−^/S486D^−^-transfected group; ^##^*P* < 0.01 vs. the PAN^+^/S486D^−^-transfected group

To investigate why the lack of SGK3 activity increased PC and ezrin ubiquitination, we tested whether the SGK3 target protein Nedd4-2, a common E3 ubiquitin ligase, was involved in podocyte damage in vivo or in vitro. We first confirmed that SGK3 regulated Nedd4-2 phosphorylation (Fig. 6a). That is, the p-Nedd4-2/Nedd4-2 ratio decreased significantly in the SGK3-K191M-transfected mouse podocytes compared to the scramble plasmid-transfected mouse podocytes. We next verified the involvement of Nedd4-2 in podocyte damage both in vivo and in vitro. The p-Nedd4-2/Nedd4-2 ratio decreased obviously in both the ADR nephritic mouse model and the PAN-induced podocyte injury cell model, implying a significant increase in Nedd4-2 activity and confirming the involvement of Nedd4-2 in podocyte damage (Fig. 6b, c). Finally, we investigated the role of the SGK3/Nedd4-2 signaling pathway in PAN-induced podocyte damage in vitro. The activity of Nedd4-2 was lower in PAN^−^/S486D^+^-transfected mouse podocytes than in PAN^−^/S486D^−^-transfected mouse podocytes (Fig. 6c). However, SGK3 activation completely reversed the PAN-induced decrease in the p-Nedd4-2/Nedd4-2 ratio (Fig. 6c). Therefore, our results strongly suggested that the SGK3/Nedd4-2 signaling pathway was involved in podocyte damage both in vivo and in vitro.

### Nedd4-2 modulated ezrin protein expression, but not PC protein expression, through the ubiquitin–proteasome pathway in cultured mouse podocytes

**Fig. 7:**
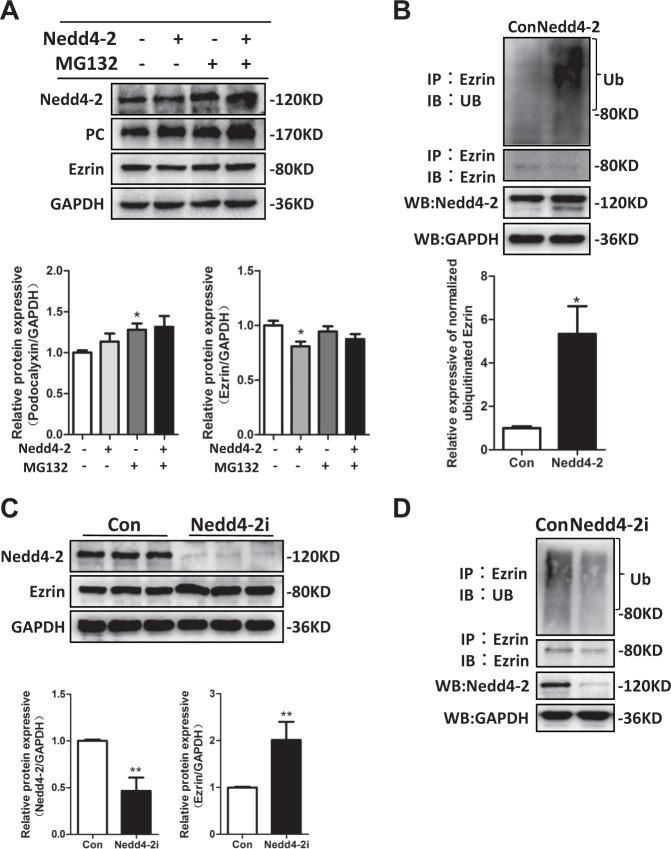
Nedd4-2 modulated ezrin ubiquitination but not PC ubiquitination. Mouse podocytes were transfected with either the pcDNA3 (Con) or the WT Nedd4-2 plasmid for 48 h. Cells were either untreated or treated with MG132 for the last 8 h of transfection. **a** Upper panel: immunoblot showing Nedd4-2, PC, and ezrin expression; GAPDH was used for normalization. Bottom panel: densitometric quantification of PC and ezrin. *P < 0.05 vs. Nedd4-2^-^/MG132^-^group. **b** WCLs were precipitated with the anti-ezrin antibody, and the immunoprecipitates were immunoblotted with anti-ezrin and anti-Ub antibodies. Upper panel: immunoblots were used to measure Nedd4-2 expression in WCLs after transfection with the WT Nedd4-2 plasmid. Bottom panel: The level of ubiquitinated ezrin was normalized to the immunoprecipitated ezrin. Data are means ± SEs of three independent experiments. *P < 0.05 vs. control group. **c** Mouse podocytes were infected with scramble (control) or Nedd4-2-specific shRNAs for 72 h. Top panel: immunoblot showing Nedd4-2 and ezrin expression; GAPDH was used for normalization. Bottom panel: densitometric quantification of Nedd4-2 and ezrin. Data are means ± SEs of three independent experiments; for each sample, three wells were treated. **P < 0.01 vs. the control group. **d** Mouse podocytes were transfected with recombinant lentiviral Nedd4-2-eGFP shRNA or scramble (control) lentiviral shRNA. WCLs were precipitated with the anti-ezrin antibody, and the immunoprecipitates were immunoblotted with anti-ezrin and anti-Ub antibodies. Nedd4-2 expression in WCLs was measured with immunoblot assays after Nedd4-2 shRNA transfection

To investigate whether the SGK3 target protein Nedd4-2 was involved in PC and ezrin expression and ubiquitination, we examined the effect of Nedd4-2 activation on PC and ezrin protein expression. Mouse podocytes were transfected with the wild-type (WT) Nedd4-2 plasmid or scramble plasmid for 48 h. Transfected mouse podocytes were then untreated or treated with MG132 for the last 8 h of transfection. Ezrin protein expression was significantly lower in the Nedd4-2^+^/MG132^−^-transfected mouse podocytes than in the Nedd4-2^−^/MG132^−^-transfected mouse podocytes, while PC protein expression remained unchanged (Fig. 7a). Moreover, after administration of MG132, the decreased expression of ezrin in the Nedd4-2^+^/MG132^−^ group was partially reversed in the Nedd4-2^+^/MG132^+^ group (Fig. 7a). Ezrin ubiquitination was significantly higher in WT Nedd4-2-transfected mouse podocytes than in the scramble plasmid-transfected mouse podocytes (Fig. 7b). These results indicated that Nedd4-2 downregulated ezrin, but not PC, through the ubiquitin–proteasome degradation pathway. All three Nedd4-2 short hairpin RNAs (shRNAs; #1, #2, and #3), as well as the Nedd4-2 control shRNA, were used to infect mouse podocytes. Although two Nedd4-2 shRNAs (#2 and #3) visibly inhibited Nedd4-2, the shRNA (#2) had the highest Nedd4-2 inhibition efficiency and was thus selected for subsequent infections (data not shown). Silencing Nedd4-2 increased ezrin protein expression and increased ezrin ubiquitination (Fig. 7c, d). Together, our results strongly suggested that Nedd4-2 downregulated ezrin protein expression by increasing ezrin ubiquitination.

### SGK3-mediated Nedd4-2 activity affected the interaction between Nedd4-2 and ezrin

**Fig. 8:**
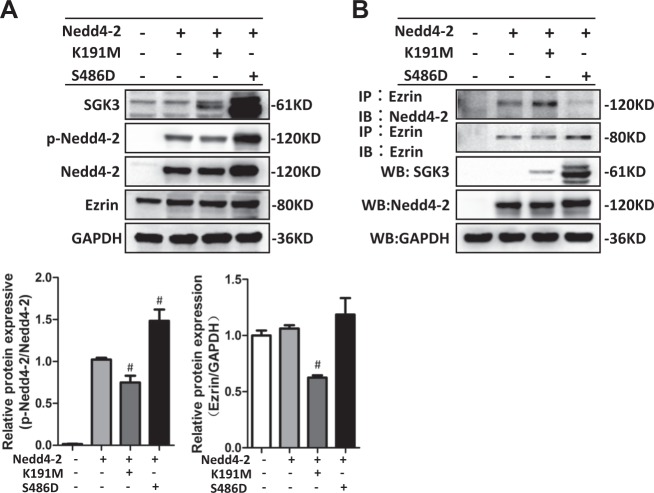
SGK3-mediated Nedd4-2 phosphorylation affected the interaction between Nedd4-2 and ezrin. **a** Upper panel: HEK293T cells were transfected with the indicated plasmids. Immunoblot analysis of SGK3, P-Nedd4-2, Nedd4-2, and ezrin expression; GAPDH was used for normalization. Bottom panel: densitometric quantification of the P-Nedd4-2/Nedd4-2 ratio and ezrin. Data are means ± SEs of three independent experiments. ^#^*P* < 0.05 vs. cells transfected with Nedd4-2^+^/K191M^−^/S486D^−^. **b** WCLs were precipitated with the anti-ezrin antibody, and the immunoprecipitates were blotted with anti-ezrin and anti-Nedd4-2 antibodies. SGK3 and Nedd4-2 expression levels in the WCLs were measured with immunoblot assays

As our results indicated that Nedd4-2 induced ezrin ubiquitination, we next tested whether SGK3-mediated Nedd4-2 activity affected the interaction between Nedd4-2 and ezrin. To test this, we performed Co-IP studies on HEK293T cells. HEK293T cells were transfected with WT Nedd4-2, with or without one of the plasmids SGK3-K191M and SGK3-S486D. Transfection with SGK3-K191M led to increased Nedd4-2 activity and decreased ezrin expression, while transfection with SGK3-S486D led to decreased Nedd4-2 activity (Fig. 8a). As ezrin harbors PY, the motif targeted by Nedd4-2, it was unsurprising that Nedd4-2 and ezrin interacted in HEK293T cells under basic conditions (Fig. 8b). After transfection with SGK3-K191M and WT Nedd4-2, Nedd4-2 and ezrin interaction increased in HEK293T cells, while Nedd4-2 and ezrin interaction decreased after transfection with SGK3-S486D and WT Nedd4-2 (Fig. 8b). Therefore, increased Nedd4-2 activity mediated by the inactivation of SGK3 also increased the interaction between Nedd4-2 and ezrin.

### SGK3 and the SGK3/Nedd4-2 signaling pathway are involved in the regulation of the ezrin/PC interaction

**Fig. 9:**
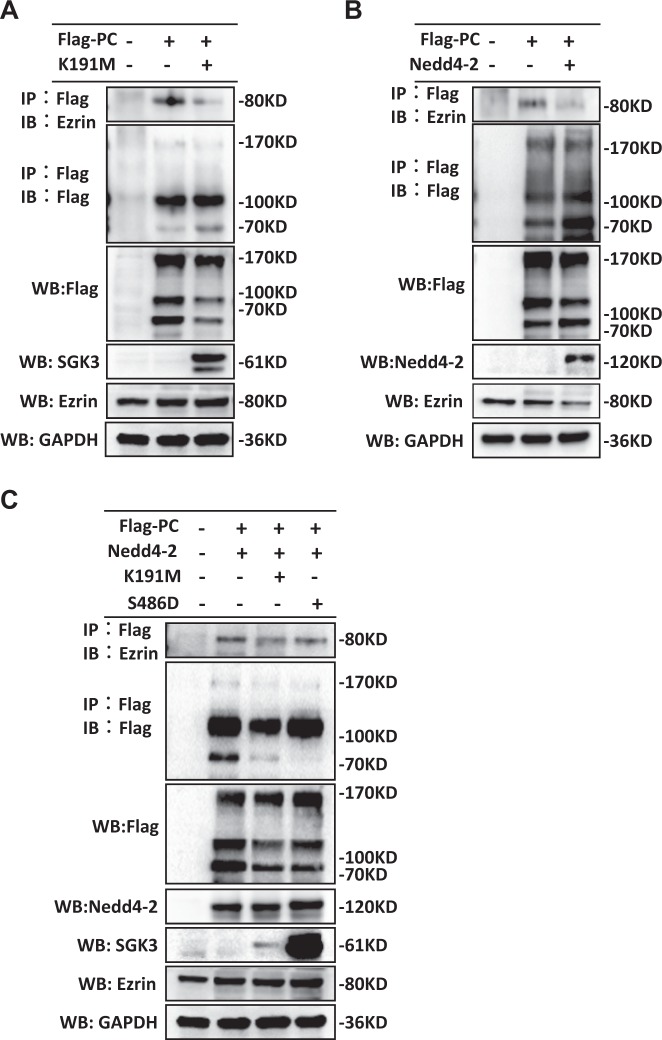
SGK3 and the SGK3/Nedd4-2 signaling pathway were involved in the regulation of PC/ezrin interaction. **a** HEK293T cells were mock transfected or transfected with the SGK3-K191M plasmid for 24 h. WCLs were precipitated with anti-FLAG antibodies and then blotted with anti-FLAG and anti-ezrin antibodies. FLAG, SGK3, and ezrin expression levels were measured in the WCLs with immunoblot assays. **b** pc-FLAG-transfected HEK293T cells were mock co-transfected or co-transfected with the WT Nedd4-2 plasmid for 24 h. Immunoblots of the WCLs were precipitated with anti-FLAG antibodies and blotted with anti-FLAG and anti-ezrin antibodies. FLAG, Nedd4-2, and ezrin expression levels in the WCLs were measured with immunoblot assays. **c** HEK293T cells were transfected with the indicated plasmids for 24 h. Immunoblots of the WCLs were precipitated with the anti-FLAG antibody, and blotted with anti-FLAG and anti-ezrin antibodies. FLAG, SGK3, Nedd4-2, and ezrin expression levels in the WCLs were measured with immunoblot assays

To explore whether SGK3 and the SGK3/Nedd4-2 signaling pathway regulated the stability of the PC/ezrin complex, we performed Co-IP assays in HEK293T cells. To do this, we first transfected the SGK3-K191M plasmid into HEK293T cells expressing PC-FLAG. We detected three bands in the PC-FLAG-transfected HEK293 cells. PC, but not ezrin, protein expression was downregulated in the transfected HEK293T cells, as compared to control cells (Fig. 9a). As Nedd4-2 is not expressed in HEK293 cells^32,33^, these results suggested that SGK3-mediated ezrin expression depended on Nedd4-2 but that SGK3-mediated PC expression did not. Moreover, the inactivation of SGK3 decreased PC expression, directly reducing the interaction between PC and ezrin (Fig. 9a).

We next transfected the HEK293T cells expressing PC-FLAG with the WT Nedd4-2 plasmid. Ezrin protein expression was downregulated after WT Nedd4-2 plasmid transfection; PC protein expression remained constant (Fig. 9b). The interaction between PC and ezrin in the cells transfected with WT Nedd4-2 was significantly lower than that in the control cells. This indicated that the activation of Nedd4-2 decreased ezrin expression, but not PC expression, contributing to the decreased interaction between PC and ezrin. In addition, we found that ezrin co-precipitated with PC in the PC-FLAG-transfected HEK293T cells, indicating that exogenously expressed PC interacts with endogenous ezrin in HEK293T cells, and suggesting that this interaction does not require the co-expression of additional podocyte-specific proteins (Fig. 9a, b).

Finally, we examined the role of the SGK3/Nedd4-2 signaling pathway with respect to the PC/ezrin interaction. To do this, HEK293T cells expressing PC-FLAG were co-transfected with WT Nedd4-2 and either the SGK3-K191M or the SGK3-S486 plasmid. The PC/ezrin interaction was reduced in cells transfected with SGK3-K191M as compared to control cells, while the PC/ezrin interaction increased in cells transfected with SGK3-S486D as compared to control cells (Fig. 9c). As SGK3 targets Nedd4-2, our results suggested that SGK3 and the SGK3/Nedd4-2 signaling pathway were both involved in regulating the stability of the PC/ezrin complex.

## Discussion

SGK3, an important downstream target protein of phosphatidylinositol-3-kinase, is involved in the regulation of substrate transport^7^, as well as cell growth, survival, and proliferation^34,35^. Previously, we showed that SGK3 deficiency leads to decreased podocin expression and reduced podocyte viability^4^. However, the signaling mechanisms involved in SGK3-triggered podocyte function are not well known. Here we found that PC and ezrin protein expression levels were reduced after podocyte damage, both in vivo and in vitro. We demonstrated that both PAN treatment and SGK3 inactivation resulted in increased PC and ezrin ubiquitination and that SGK3 activation partially reversed PAN-induced decreases in PC and ezrin protein expression. Moreover, the enhancement of Nedd4-2 activity via SGK3 inactivation increased ezrin ubiquitination but not PC ubiquitination. Our results suggested that SGK3 and the SGK3/Nedd4-2 signaling pathway helped to maintain the stability of the PC/ezrin complex in vitro.

One intriguing finding of our study was that decreased PC and ezrin protein expression was not caused by reduced PC and ezrin mRNA transcription after PAN-induced podocyte damage. Rather, this decreased protein expression primarily resulted from the increased PC and ezrin protein degradation induced via the ubiquitinproteasome pathway. Ezrin, a plasma membrane–cytoskeleton linker protein, mainly regulates cell adhesion and morphogenesis^36^. Previous studies have shown that ezrin activity and/or expression decreases in animal models of glomerular disease^28,37^ and in nephropathic patients^29,37^. Consistent with these studies, we found that ezrin was downregulated after podocyte damage both in vivo and in vitro, suggesting that decreased ezrin expression may be a marker for podocyte damage. PC is the most highly sialylated glycoprotein in podocytes^13,23^. Several studies have reported the downregulation of PC in ADR models^38–40^ and upregulation of PC mRNA expression in mouse podocytes after PAN-induced podocyte damage^41,42^. Both of these findings were consistent with our results. Incongruent with our results, some studies have shown that PC protein expression increased in mouse podocytes with PAN-induced podocyte damage^41,42^, but this discrepancy was probably due to differences in experimental methods (e.g., PAN treatment period or concentration).

We previously implicated SGK3 in the mediation of PAN-induced podocyte damage^4^, but the mechanisms underlying this mediation remained unclear. Here we linked SGK3 activation to PC and ezrin protein expression after podocyte damage, both in vivo and in vitro. The downregulation of SGK3 not only suppressed PC mRNA transcription but also inhibited PC and ezrin protein expression. In addition, SGK3 activation mostly reversed the PAN-induced inhibition of PC and ezrin protein expression. Our results therefore convincingly indicated that SGK3 affected PAN-induced podocyte damage by triggering PC and ezrin protein expression. It should be noted that SGK3 inactivation decreased PC mRNA expression, while PAN treatment increased PC mRNA expression, suggesting that PAN-induced podocyte damage did not depend solely on SGK3 activity. The mechanisms underlying this contradiction (i.e., that PAN treatment led both to SGK3 inactivation and to increased PC mRNA expression) are still unclear and merit further investigation.

We have shown here, for the first time, that SGK3 activation triggers the ubiquitin-mediated degradation of PC and ezrin. This novel result provides insight into the pathomechanism of SGK3 in podocytes. It is well known that PC and ezrin are regulated by a variety of factors, including phosphorylation, binding partners^28^, and ubiquitination^43^. A recent study showed that ezrin was ubiquitinated by WWP1, a HECT ubiquitin ligase^26^ that mediates the alteration of ezrin function, but does not degrade ezrin^44^. However, the ubiquitination of PC has not previously been reported. Herein we found that SGK3 inactivation led to PC and ezrin protein degradation, primarily via the proteasome pathway. Interestingly, we found that the SGK3-targeted protein Nedd4-2 mediated ezrin ubiquitination, but not PC ubiquitination, indicating that SGK3 modulates PC and ezrin degradation through different regulatory mechanisms. This result was consistent with the known protein structures, in that there is a PY motif in ezrin but not in PC. The PY motif can be bound, and therefore regulated, by Nedd4-2. Consistent with this, SGK3 phosphorylates Nedd4-2 and regulates the binding of Nedd4-2 with its target protein, ezrin (Fig. 8). It should be stressed that the molecular details of SGK3-mediated PC ubiquitination remain obscure. In addition, unlike WWP1-mediated ezrin ubiquitination, Nedd4-2-mediated ezrin ubiquitination decreased ezrin protein expression.

Several studies have shown that structural and/or functional defects in either PC or the associated cytoskeletal linker proteins may result in glomerular disorders^17^. Disruption of the PC/ezrin interaction in glomerular epithelial cells is associated with the dephosphorylation of ezrin, the neutralization of the negative PC surface charge^17,24^, and the decreased protein expression of either PC or ezrin^24^. Here we found that the deceased SGK3 activity resulted in decreased PC and ezrin protein expression, consistent with the disrupted binding of PC and ezrin in SGK3-inactivated mouse podocytes and HEK293T cells. SGK3 downregulation led to decreased PC mRNA expression and to increased PC and ezrin protein degradation. Together, these effects might disrupt the PC/ezrin interaction. Notably, Nedd4-2 activity did not influence PC expression. This indicated that the decreased stability of the PC/ezrin complex induced by the decrease in SGK3 activity was not completely dependent on the SGK3/Nedd4-2 signaling pathway. Several studies have focused on the role of AKT in the kidney^8–10^. AKT is highly structurally and functionally homologous with SGK3^11^. AKT2 maintains podocyte viability and function via the AKT/mammalian target of rapamycin or the AKT/GSK3β pathways^10,45^. SGK3 might regulate PC expression through the similar AKT pathway, and indeed, other pathways underlying podocyte dysfunction might be induced by a decrease in SGK3 activity.

**Fig. 10:**
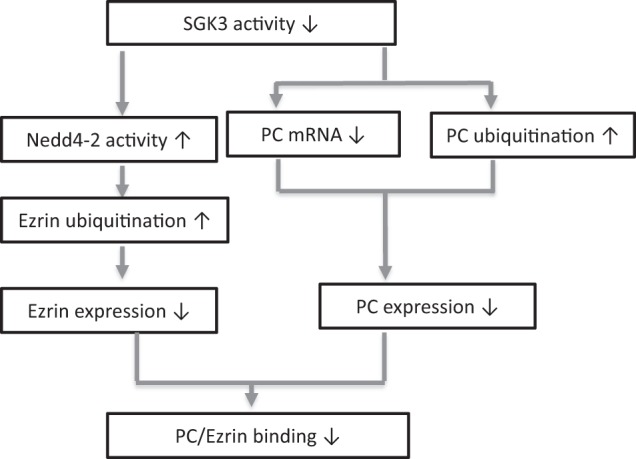
Schematic diagram showed how SGK3 stabilizes the PC/ezrin complex. SGK3 inactivation upregulated the activity of its target protein Nedd4-2, which resulted in increased ezrin ubiquitination, downregulated ezrin protein expression, and reduced PC and ezrin interaction. In addition, downregulated SGK3 activity also reduced PC mRNA and protein expression, thus affecting the stability of the PC/ ezrin complex

In summary, SGK3 inactivation played an important role in the regulation of PC and ezrin expression. Here we demonstrated that SGK3 inactivation downregulated PC mRNA and upregulated the ubiquitin-mediated degradation of PC and ezrin, thereby inhibiting the protein expression of PC and ezrin. These changes destabilized the PC/ezrin complex. Moreover, SGK3-triggered Nedd4-2 activity degraded ezrin rather than PC (Fig. 10). Collectively, our results suggested that the regulation of SGK3 and the SGK3/Nedd4-2 signaling pathway determines the stability of the podocyte PC/ezrin complex.

## Methods and materials

### Reagents and antibodies

Polyclonal rabbit anti-SGK3, anti-Nedd4-2 and anti-phospho-Nedd4-2 (Ser342) antibodies were purchased from Cell Signaling Technology, Inc. (Beverly, MA, USA). Polyclonal rabbit anti-ezrin was purchased from Abcam (Cambridge, MA, USA). Polyclonal goat anti-PC antibody was purchased from Research & Diagnostics Systems, Inc. (Minneapolis, MN, USA). Polyclonal rabbit anti-Desmin (I-448) was purchased from Bioworld Technology, Co. Ltd. (Nanjing, China). Monoclonal mouse anti-GAPDH antibody; polyclonal rabbit anti-flag tag antibody; and horseradish peroxide-conjugated anti-rabbit, anti-mouse, and anti-goat secondary antibodies were purchased from Proteintech (Wuhan, China). Monoclonal mouse anti-ubiquitin antibody was purchased from Covance Inc. (UT, USA). The pcDNA3/mNedd4-2, pMO/mSGK3-K191M (SGK3-K191M), and pMO/mSGK3-S486D (SGK3-S486D) plasmids were constructed as previously described^4^. The PC-Myc-DDK plasmid (Flag-epitope at N^−^terminal of mouse PC) was purchased from Origene Technologies, Inc. (Rockville, MD, USA). The plasmid of the mouse Flag-tagged WT Ub was provided by Dr. Qiuhong Duan (Department of Biochemistry and Molecular Biology, Tongji Medical College, Huazhong University of Science & Technology, Wuhan, China). Three recombinant lentiviruses against Nedd4-2 were designed and generated by Genechem (Shanghai, China). PAN was purchased from Sigma Chemical (St Louis, MO, USA). MG132 was purchased from Selleck (Shanghai, China). A/G plus agarose for immunoprecipitation (IP) assay was purchased from Beyotime Biotechnology (Shanghai, China). Phosphatase inhibitor mixture and proteinase inhibitor cocktail were purchased from Roche Applied Science (Indianapolis, IN).

### ADR nephritis mouse model

All experiments were performed in accordance with the National Institutes of Health guidelines for the use and care of experimental animals and were approved by the Animal Care and Use Committee of Tongji Medical College. The generation and characters of ADR-induced nephrosis model were previously described^4^. Briefly, male mice (8-week-old), body weight 20–25 g, were treated with a single dose of 10.5 mg/kg ADR (Sigma, USA, D1515) by tail vein injection (ADR group, *n* = 6). The mice in the control group were administered with an equivalent volume of phosphate-buffered saline (PBS) (control group, *n* = 6). On day 14, the mice were sacrificed under anesthesia and both blood samples and kidneys were collected. The harvested kidneys were collected for western blotting and immunohistochemical analysis.

### Histochemistry and immunohistochemistry

PAS staining and immunohistochemistry studies proceeded and analyzed essentially as previously described^4^. In short, paraffin-embedded mouse kidney sections (5-μm thickness) for PAS and immunohistochemical staining were performed using routine protocol. Sections were stained with PAS reagent by standard procedure. Immunohistochemistry staining was performed using routine protocol. The slides were incubated with rabbit polyclonal anti-ezrin antibody and goat anti-PC antibody overnight at 4 °C, followed by incubation with poly-peroxidase anti-rabbit and anti-goat IgG secondary antibodies for 30 min at room temperature. Staining was then performed using a DAB Kit. Histological examination was performed via light microscopy. Positive expression for PC and ezrin were semi-quantitative using the Image Pro Plus software.

### Urine and serum biochemistry

Blood urea nitrogen and urinary and serum creatinine levels were determined by using the respective kits (NINGBO ELEJECH Biological Technology Co., LTD, Zhejiang, China), according to the manufacturer’s protocols. Urinary albumin was standardized to urinary creatinine and expressed as mg/mg Ucr.

### Cell culture, transfection, and recombinant lentivirus infection

The conditional immortalized mouse podocytes were handled as described earlier^4^. Briefly, cells were maintained in RPMI 1640 medium (HyClone, USA) supplemented with 10% (v/v) fetal bovine serum (GIBCO, Thermo Fisher Scientific Inc. USA), 100 U/ml penicillin, and 100 µg /ml streptomycin. To propagate podocytes, cells were cultivated on type I collagen at 33 °C under 5% CO_2_ and 95% air in culture medium supplemented with 10 U/ml recombinant interferon-γ (IFN-γ). To induce differentiation, podocytes were transferred at 37 °C in IFN-γ-free medium. Mouse podocytes between passages 9 and 20 were used in all experiments. HEK293T cell cultures were maintained at 37 °C in Dulbecco’s modified Eagle’s medium (HyClone, USA), supplemented with 10% fetal bovine serum, 100 U/ml penicillin, and 100 μg/ml streptomycin. Mouse podocytes and HEK293T cells were transiently transfected with various plasmids and respective scramble plasmids using Lipofectamine 2000 reagent (Invitrogen, Carlsbad, CA) according to the manufacturer’s protocol for adherent cells. Cells were analyzed 24–48 h post transfection. Mouse podocytes were infected with scramble or Nedd4-2-specific lentiviruses. Seventy-two hours after shRNA infection, western blotting was used to evaluate the suppression of Nedd4-2 expression in different cell groups.

### Cell viability assay

Cell viability was assessed by an MTT assay. Briefly, mouse podocytes were seeded into 96-well plates at a density of 2 × 10^3^/well followed by treatment with or without PAN administration for 24 h. In all, 10 μl of 5 mg/ml MTT solution in PBS was added without removal of culture medium followed by incubation at 37 °C for 4 h. The medium was removed and 100 μl of dimethyl sulfoxide was added to dissolve the formazan crystals for about 15 min by a shaker. Absorbance of each well was measured by a microplate reader (BioTek, Winooski, VT, USA) at 570-nm wavelength, followed by calculating the mean value. All data were determined from three independent cultures.

### Immunofluorescence staining and and confocal microscopy

Mouse podocytes grown on glass coverslips were fixed with 4% paraformaldehyde for 30 min and permeabilized with 0.1% Triton X-100 in PBS for 5 min. After blocking with 10% donkey serum for 30 min, the slides were immunostained with primary antibodies against PC and ezrin. The slides were then stained with IFKine green-conjugated donkey anti-goat antibody (#A24231, Abbkine) and Alexa Fluor 594-conjugated donkey anti-rabbit antibody (127803, Jackson). Cells were costained with 4,6-diamidino-2-phenylindole (blue). For F-actin staining, slides was stained with rhodamine–phalloidin (Invitrogen, Carlsbad, CA) for 20 min at room temperature. Images were acquired using a Leica TCS-SL confocal microscope.

### Western blot analysis

Protein expression was analyzed by western blot analysis as described previously^4^. In brief, protein extracts from the kidneys as well as whole-cell protein from stable cell lines were used. Equal amounts of proteins were loaded on sodium dodecyl sulfate-polyacrylamide gel electrophoresis (SDS-PAGE) gels for separation and transferred onto polyvinylidene difluoride membrane (Millipore Corp., Bedford, MA). Membranes were then blocked for 1 h at room temperature using 5% skim milk and 0.1% Tween 20 in Tris-buffered saline. The immunoblot was then performed overnight at 4 °C using appropriate primary antibodies. Protein signals were detected using the corresponding horseradish peroxidase-conjugated secondary antibody and enhanced chemiluminescence (Millipore, Germany). All experiments were performed in triplicate, and the blots of p-Nedd4-2 and Nedd4-2 were taken from the same membrane.

### Real-time RT-PCR analysis

Total RNA isolation and quantitative RT-PCR (qRT-PCR) were carried out by a routine procedure. Total RNA was isolated from incubated cells using TRIzol Reagent (Invitrogen, Thermo Fisher Scientific Inc. USA) according to the manufacturer’s instructions. The first-strand cDNA was carried out using a ReverAid First Strand cDNA Synthesis Kit (Thermo, Thermo Fisher Scientific Inc. USA) according to the manufacturer’s protocol. Real-time RT-PCR was performed on the CFX96 Touch™ Real-Time PCR Detection System (Bio-rad, Hercules, CA, USA). The PCR results for each sample were normalized using glyceraldehyde 3-phosphate dehydrogenase (GAPDH) mRNA as an internal control. The ΔΔCt technique was used to calculate cDNA content in each sample. Primers for PC and ezrin were designed based on the GenBank accession numbers (PC: NM_013723, ezrin: NM_22350). The sequences of the primer pairs used in RT-PCR are as follows: mouse PC forward: CTGTAAGCGACCTGAACCGT, backward: TGCTGCCACCTTGATGTCTA; mouse ezrin forward: TCACAGAGGCAGAGAAGAATG, backward: TCTCGTTGTGGATGATGTCA; and mouse GAPDH forward: GTCAAGCTCATTTCCTGGTATG, backward: CCTCTCTTGCTCAGTGTCCTT. All experiments were performed in triplicate.

### Ubiquitination assay in mouse podocytes

Ubiquitination assays were performed as described^32^. Mouse podocytes were treated with 50 μg/ml PAN or transfected with expression constructs (as shown in the figures). To inhibit proteasomal degradation, 10 μM MG132 was added to mouse podocytes 8 h before lysis. Twenty-four hours after PAN treatment or 48 h after transfection, cells were washed with PBS and dissociated in 1 ml dissociation buffer [5% glycerol, 1 mM EDTA, and 1 mM EGTA (pH 8.0)]. Then cells were washed with 10 ml PBS and centrifuged at 4 °C, 3000 rpm, for 15 min. Cell pellets were frozen at −70 °C for 2–4 h. Pellets were then resuspended in 1 ml lysis buffer [50 mM HEPES (pH 7.5), 1 mM EGTA, 150 mM NaCl, 1.5 mM MgCl2, 10% glycerol, 1% TritonX-100, 25 mM sodium fluoride, 40 mM β-glycerolphosphate, 10 mM sodium pyrophosphate] containing phenylmethanesulfonylfluoride, cocktail, and phosphatase inhibitors. The lysates were centrifuged at 13,000 rpm (4 °C) for 15 min. The supernatant proteins (about 1.5 mg) were subjected to IP using the appropriate primary antibody (anti-ezrin antibody, anti-Flag antibody) and protein A/G agarose beads overnight at 4 °C. Sample loading buffer 5× was added to the immunoprecipitates and boiled for 10 min. The samples, centrifuged at 13,000 rpm for 5 min, were separated by SDS-PAGE for western blot analysis and immunoblotted with anti-ubiquitin or anti-PC antibody. Scanning densitometry was measured as described above.

### Co-IP assays

Mouse podocytes were maintained and administered with PAN for 24 h or transfected with appropriate expression constructs for 48 h. HEK293T cells, grown on 10-cm dishes, were transfected with 10 μg of the plasmids DNA encoding FLAG-tagged PC, mouse Nedd4-2, mouse SGK3-S486D, mouse SGK3-K191M, and/or empty vector using Lipofectamine 2000 reagent for 24 h. After treatment and transfection, cells were dissociated in 500 μl IP lysis buffer (Beyotime Biotechnology, Shanghai, China). Proteins (about 1.0 mg) were incubated with the appropriate primary antibody (anti-ezrin antibody, anti-Flag antibody) and protein A/G agarose beads. Then the samples were subjected to western blot analysis.

### Statistical analysis

All data are expressed as the mean ± SE. SPSS 13.0(SPSS, Inc., Chicago, IL) was used for data analysis. A one-way analysis of variance (ANOVA) or two-tailed Student’s *t* test was used to determine statistical significance between the control and test groups. A one-way ANOVA with Dunnett’s posttest for >2 groups. A *P* value of ≤0.05 was considered significant. Analyses were performed using the Graphpad Instat software (San Diego, CA).
