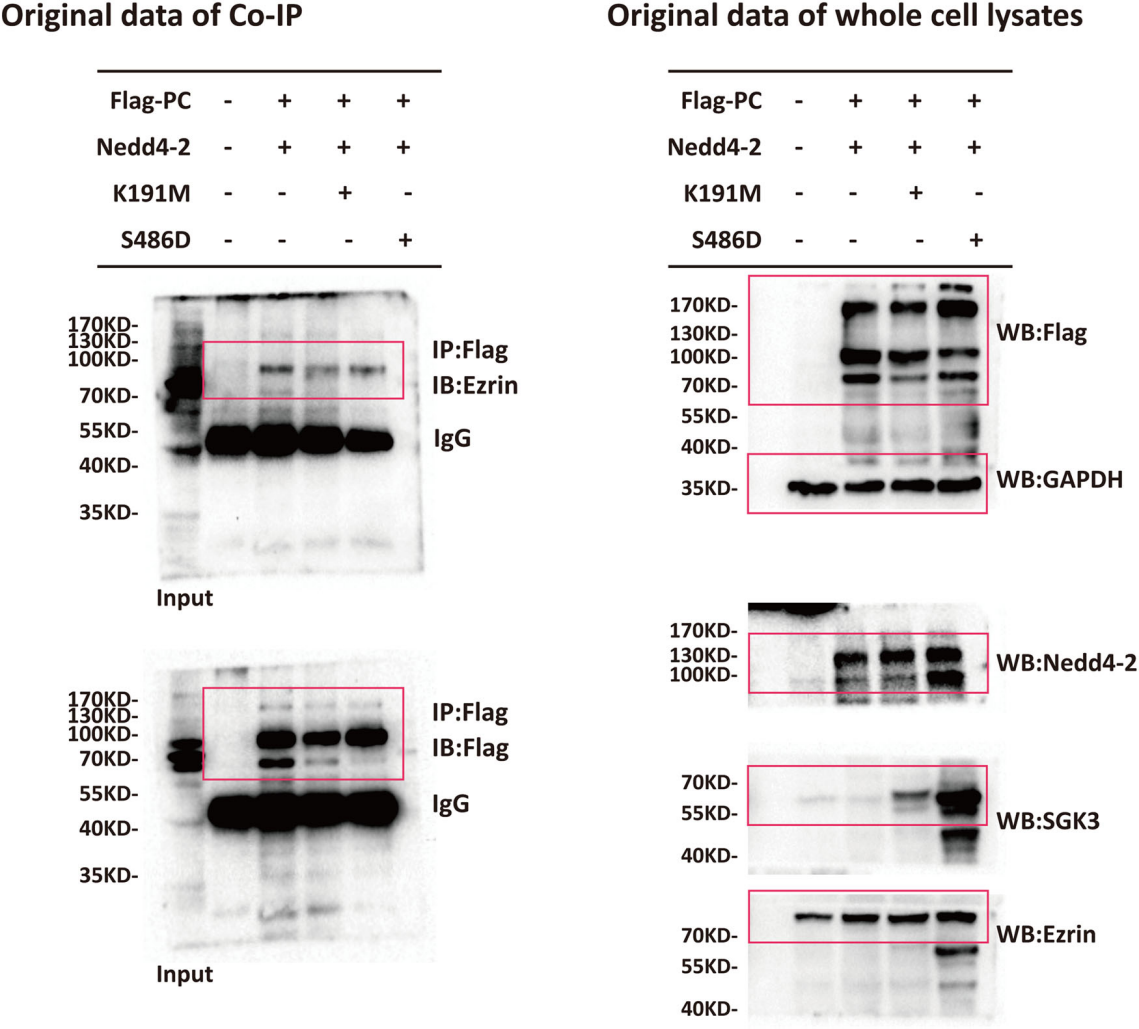# Correction: The SGK3-triggered ubiquitin-proteasome degradation of podocalyxin (PC) and ezrin in podocytes was associated with the stability of the PC/ezrin complex

**DOI:** 10.1038/s41419-025-07369-7

**Published:** 2025-04-03

**Authors:** Ya-Pei Yuan, Hong Zhao, Li-Qin Peng, Zi-Fang Li, Song Liu, Cheng-Yan Yuan, Mercy-Julian Mwamunyi, David Pearce, Li-Jun Yao

**Affiliations:** 1 Department of Nephrology, Union Hospital, Tongji Medical College, Huazhong University of Science and Technology, 430022 Wuhan, China; 2 Department of Trauma Surgery, Tongji Hospital, Tongji Medical College, Huazhong University of Science and Technology, 430030 Wuhan, China; 3 Department of Rheumatology, Sun Yat-sen Memorial Hospital, Sun Yat-sen University, 510120 Guangzhou, China; 4 Department of Medicine, University of California, San Francisco, CA 94107-2140 USA; 5 Department of Molecular and Cellular Pharmacology, University of California, San Francisco, CA 94107-2140 USA

Correction to: *Cell Death and Disease* 10.1038/s41419-018-1161-1, published online 01 November 2018

In the original version of the Article, the blots of ezrin and GADPH were misplaced in Figure 9C during figure assembly, resulting in duplication with Figure 8C. We provided a revised version with original data.

The authors apologize for this error and state that this does not change the scientific conclusions of the article in any way.

Updated version of Fig. 9C
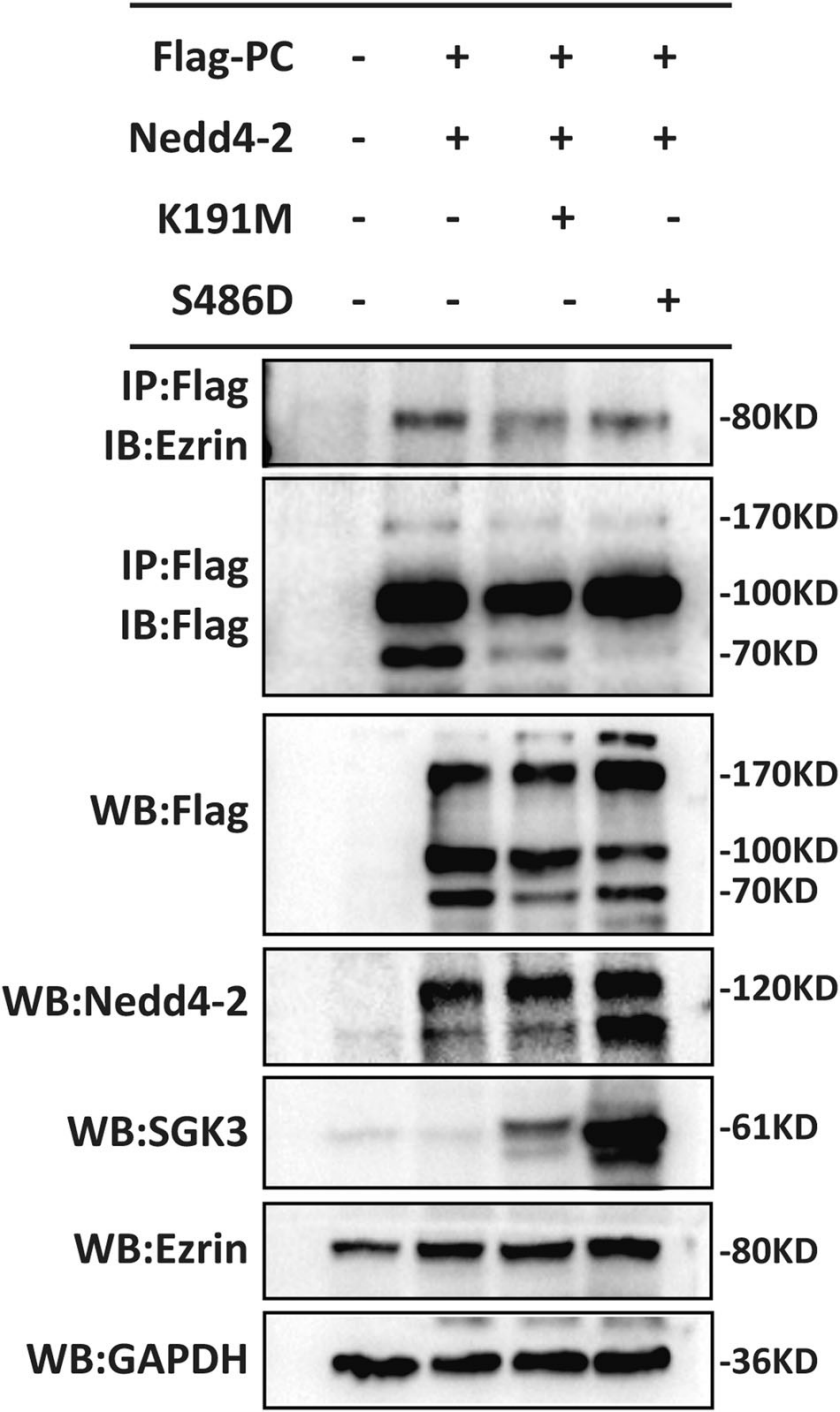


Original data of updated version of Fig. 9C